# *Acinetobacter* spp. in Gunshot Injuries

**DOI:** 10.3201/eid1401.070878

**Published:** 2008-01

**Authors:** James W.T. Elston, Ciaran L. Bannan, Desmond T. Chih, Craig S. Boutlis

**Affiliations:** * Castle Hill Hospital, Cottingham, East Yorkshire, United Kingdom; † Royal Darwin Hospital, Darwin, Northern Territory, Australia; ‡ Charles Darwin University, Darwin, Northern Territory, Australia

**Keywords:** Acinetobacter infections, drug resistance, multiple, bacterial, wounds, gunshot, wound infection, osteomyelitis, East Timor, letter

**To the Editor:** Challenges posed by *Acinetobacter* spp. result from multidrug resistance, nosocomial spread, and hospital-wide outbreaks (*1*–*3*). We evaluated *Acinetobacter* spp. infections from gunshot injuries received during the April 2006 East Timor conflict (for a description of these events and further reading, see http://en.wikipedia.org/wiki/2006_east_timorese_crisis).

We reviewed records of 15 injured East Timorese police officers. Median age was 29 years (range 25–45 years); 13 were male. Typical injuries were from multiple high-velocity gunshots and shrapnel. All patients had undergone surgery for stabilization and wound debridement before evacuation to the Royal Darwin Hospital (RDH) in Australia; most had likely received antimicrobial drugs including ampicillin, gentamicin, metronidazole, and ceftriaxone. They arrived at RDH a median of 3 days after injury (range 2–12 days).

The patients were separated from other hospital inpatients on arrival; they were managed as a cohort, they had dedicated nursing staff, and barrier contact precautions were practiced. However, the patients were not routinely screened for colonization with microbiologic organisms. Additional surgical management, including further wound debridement, was performed on 12 of the 15 patients (11 within 48 hours of arrival at RDH); intraoperative samples of bone, soft tissue, and wounds were submitted for culture.

From 13 patients (including all 11 with gunshot wounds), 19 *Acinetobacter* spp. isolates were recovered. *Acinetobacter* spp. was cultured from deep wound tissue obtained during surgery from 9 patients. Substantial antimicrobial drug resistance was demonstrated by automated testing (Vitek 2, bioMérieux, Marcy l’Etoile, France) (Table). All 19 *Acinetobacter* spp. isolates were classified as multidrug resistant (resistant to >3 drug classes) (*4*). Isolates from 10 of the 13 culture-positive patients (12 of 19 isolates) were resistant to all tested drugs except meropenem and amikacin. Susceptibility testing for tigecycline and tetracycline was not performed. No isolate was metallo-β-lactamase positive by phenotypic analysis according to tablet disk diffusion method using imipenem and imipenem plus EDTA Neo-Sensitabs (Rosco Diagnostica, Taastrup, Denmark). Isolation of *Acinetobacter* spp. (15 isolates) far exceeded that of other organisms: *Stenotrophomonas* (5 isolates); *Pseudomonas aeruginosa* (3 isolates); *Staphylococcus aureus* and *Enterococcus* spp. (2 isolates each); and *Pseudomonas putida*, *Enterobacter cloacae*, *Staphylococcus hemolyticus*, and *Mycoplasma hominis* (1 isolate each).

**Table:** Susceptibility of *Acinetobacter* spp. isolates to antimicrobial drugs, % (n = 19)*

Drug	Susceptible	Intermediate	Resistant
Amikacin	100	0	0
Ampicillin	0	0	100
Ceftazidime	21	26	53
Ceftriaxone	0	0	100
Ciprofloxacin	26	16	58
Gentamicin	21	0	79
Meropenem	100	0	0
Piperacillin/tazobactam	26	32	42
Ticarcillin/clavulanic acid	21	21	58
Tobramycin	21	16	63
Trimethoprim/sulfamethoxazole	11	0	89

*Isolates from gunshot wounds of 15 persons injured in East Timor, 2006.

On the basis of clinical assessment by the treating surgeon and infectious diseases physician, 11 patients were treated for *Acinetobacter* spp. infection. Patients 1–5 had comminuted compound fractures associated with intraoperative deep wound tissue that was culture positive for *Acinetobacter* spp. and were treated for osteomyelitis; patients 6–11 were treated for wound infections; patients 6–8 had intraoperative deep wound tissue culture positive for *Acinetobacter* spp.; patients 9–10 had superficial wound swabs that were culture positive; and patient 11 had a positive culture from a nonsurgical site. Of these 11 patients, 4 had fever >38°C on the day of admission to RDH (2 of whom had a leukocyte count >20,000/μL), and another 2 had visible pus, necrotic tissue, or both. The surgical approach to these patients involved delayed wound closure; fracture fixation; vacuum dressings; and skin, bone, and nerve grafts. Choice and duration of antimicrobial drug therapy was guided by susceptibility testing and experience (*4*). Presumed osteomyelitis caused by multidrug-resistant (MDR) *Acinetobacter* spp. was treated with meropenem in combination with amikacin for at least 2 weeks, followed by another 2 weeks of meropenem monotherapy. Wound infections were similarly treated with combination therapy initially, but amikacin was stopped earlier. No aminoglycoside toxicity was observed. Treatment was stopped at 4 weeks if no signs of infection were present (healed wound plus apyrexia and a C-reactive protein level <20 mg/L). Patient 12 was colonized with MDR *Acinetobacter* spp. and was treated for aspiration pneumonia; patient 13 had MDR *Acinetobacter* spp. colonization of a central venous catheter.

Follow-up after completion of therapy ranged from 4 to 23 weeks. No patients had recurrence of infection or isolation of *Acinetobacter* spp. Defining osteomyelitis and wound infection caused by *Acinetobacter* spp. was problematic for clinicians (*4**–**6*), and some assumed infections may have represented colonization. Because treatment for MDR *Acinetobacter* spp. in this setting can be protracted and toxic (e.g., from aminoglycosides), our review highlights the potential benefits of applying prospectively documented criteria such as abnormal bone histologic findings for osteomyelitis and a workable definition of deep tissue infection to better guide treatment decisions.

RDH had not experienced outbreaks of healthcare-associated infection or colonization with MDR *Acinetobacter* spp. before or after (as of January 1, 2007) the 2006 East Timor conflict, except for positive isolates from 5 patients evacuated from the Bali bombings of 2002 and 2005. Because all but 1 isolate were recovered within 48 hours of admission, primary inoculation of *Acinetobacter* spp. into wounds is assumed to have occurred either at the time of injury (from environmental sources or preexisting skin colonization), from nosocomial transmission in East Timor (before transfer to RDH), or during evacuation to RDH. Environmental and patient-based screening at sites of primary care may help resolve the uncertainty of which source is most likely.
